# Genetic Variability of TCF4 in Schizophrenia of Southern Chinese Han Population: A Case-Control Study

**DOI:** 10.3389/fgene.2019.00513

**Published:** 2019-05-28

**Authors:** Jingwen Yin, Dongjian Zhu, You Li, Dong Lv, Huajun Yu, Chunmei Liang, Xudong Luo, Xusan Xu, Jiawu Fu, Haifeng Yan, Zhun Dai, Xia Zhou, Xia Wen, Susu Xiong, Zhixiong Lin, Juda Lin, Bin Zhao, Yajun Wang, Keshen Li, Guoda Ma

**Affiliations:** ^1^Department of Psychiatry, Affiliated Hospital of Guangdong Medical University, Zhanjiang, China; ^2^Department of Neurology, Affiliated Hospital of Guangdong Medical University, Zhanjiang, China; ^3^Guangdong Key Laboratory of Age-Related Cardiac and Cerebral Diseases, Guangdong Medical University, Zhanjiang, China; ^4^Experiment Animal Center, Guangdong Medical University, Zhanjiang, China; ^5^Clinical Research Center, Affiliated Hospital of Guangdong Medical University, Zhanjiang, China; ^6^Department of Neurology and Stroke Center, The First Affiliated Hospital, Jinan University, Guangzhou, China; ^7^Clinical Neuroscience Institute of Jinan University, Guangzhou, China

**Keywords:** schizophrenia, TCF4, polymorphisms, positive psychotic symptoms, Southern Chinese Han population

## Abstract

**Objective:** Schizophrenia is thought to be a neurodevelopmental disorder. As a key regulator in the development of the central nervous system, transcription factor 4 (TCF4) has been shown to be involved in the pathogenesis of schizophrenia. The aim of our study was to assay the association of TCF4 single nucleotide polymorphisms (SNPs) with schizophrenia and the effect of these SNPs on phenotypic variability in schizophrenia in Southern Chinese Han Population.

**Methods:** Four SNPs (rs9960767, rs2958182, rs4309482, and rs12966547) of TCF4 were genotyped in 1137 schizophrenic patients and 1035 controls in a Southern Chinese Han population using the improved multiplex ligation detection reaction (iMLDR) technique. For patients with schizophrenia, the severity of symptom phenotypes was analyzed by the five-factor model of the Positive and Negative Symptom Scale (PANSS). Cognitive function was assessed using the Brief Assessment of Cognition in Schizophrenia (BACS) scale.

**Results:** The results showed that the genotypes and alleles of the three SNPs (rs2958182, rs4309482, and rs12966547) were not significantly different between the control group and the case group (all *P* > 0.05). rs9960767 could not be included in the statistics for the extremely low minor allele frequency. However, the genotypes of rs4309482 shown a potential risk in the positive symptoms (*P* = 0.04) and excitement symptoms (*P* = 0.04) of the five-factor model of PANSS, but not survived in multiple test correction. The same potential risk was shown in the rs12966547 in positive symptoms of the PANSS (*P* = 0.03).

**Conclusion:** Our results failed to find the associations of SNPs (rs2958182, rs4309482, and rs12966547) in TCF4 with schizophrenia in Southern Chinese Han Population.

## Introduction

Schizophrenia is thought to be a highly heritable disease with a genetic architecture arising from the subtle effect of multiple risk genes (Wang et al., 2017). The “accumulation” of dysregulation events in susceptibility genes leading to dysfunction in the nervous system results in the phenotypic heterogeneity of schizophrenia, including positive symptoms, negative symptoms, and cognitive dysfunction (Takahashi, 2013).

TCF4, a transcription factor involved in the development of the nervous system, is found to be a highly plausible candidate for contributing to schizophrenia (Lennertz et al., 2011). *In vitro* and *in vivo* evidence demonstrated the involvement of TCF4 in all stages of brain development, including proliferation, differentiation, migration and synaptogenesis, as well as in adult brain plasticity and information signaling (D’Rozario et al., 2016). Rare TCF4 mutations led to neurodevelopmental disorders, such as Pitt–Hopkins syndrome, which is characterized by severe cognitive deficit, microcephaly, disrupted motor development, and hyperventilation (Forrest et al., 2012). In animal studies, overexpression of TCF4 in transgenic mice resulted in deficits in prepulse inhibition (PPI) and memory, fear conditioning and sensorimotor gating (Brzózka et al., 2010). Functional deficit in the neural system might be involved in the etiology of schizophrenia. Corresponding results in clinical studies suggested a critical effect of TCF4 in several phenotypes of schizophrenia, including age at onset, sensorimotor gating, negative symptoms, cognitive function and MRI-measured brain structure (Albanna et al., 2014; Hui et al., 2015; Chow et al., 2016; Alizadeh et al., 2017). Further analysis showed increased TCF4 mRNA expression in psychosis patients compared with controls (Wirgenes et al., 2012). Furthermore, as a basic helix-loop-helix (bHLH) transcription factor, TCF4 is considered a crucial player in gene expression networks through regulation of gene expression (Forrest et al., 2014). In particular, TCF4 has been identified as a direct target of schizophrenia-associated pivotal factor miR-137, suggesting a particular susceptibility whereby TCF4 could be involved in the gene regulatory networks underlying schizophrenia (Yin et al., 2014; Xia et al., 2018).

Large genome-wide association studies (GWAS) with some replicable and intriguing findings have suggested that several single nucleotide polymorphisms (SNPs) of TCF4 are consistent with and significantly increase susceptibility to schizophrenia (Schizophrenia Psychiatric Genome-Wide Association Study [GWAS] Consortium, 2011; Steinberg et al., 2011; Zammit et al., 2014). The variant rs9960767, located in intron 3 of the TCF4 gene on chromosome 18q21.1, was significantly associated with schizophrenia in the European population (Steinberg et al., 2011). However, it was not polymorphic in a previous case-control study in the East Chinese Han Population (Li et al., 2010). Interestingly, the rs2958182 polymorphism was predicted to be a proxy SNP for rs9960767, with a high linkage disequilibrium (LD) (*D’* = 1) and the physically (∼6 kb pairs) closest distance between two SNPs. rs2958182 has been reported to be involved in several phenotypes of schizophrenia in the Chinese population. Another risk SNP identified by Steinberg et al. (Steinberg et al., 2011), rs4309482, lies intergenically downstream of TCF4 and upstream of coiled-coiled domain containing 68 (CCDC68). Finally, in another mega-GWAS analysis, rs12966547, which was in high LD with rs4309482, was associated with the risk of schizophrenia (Schizophrenia Psychiatric Genome-Wide Association Study [GWAS] Consortium, 2011).

Considering the potential for ethnic and geographic heterogeneity, the susceptibility associated with rs9960767 and rs2958182 should be further verified in the Southern Han population in China. For the first time, we conducted a case-control study to explore the risk associated with rs4309482 and rs12966547 in schizophrenia. The effects of a genetic risk variant on phenotype, including demographic characteristics and neurocognitive function, were examined to further identify the genetic sources of phenotypic heterogeneity.

## Materials and Methods

### Ethics Statement

The study was approved by the Ethical Committee of the Affiliated Hospital of Guangdong Medical University, and written consent forms were obtained from the participants or their legal representatives.

### Subjects

A total of 1137 unrelated patients with schizophrenia were consecutively recruited from the Affiliated Hospital of Guangdong Medical University. All patients were enrolled based on the following criteria: (1) diagnosed as schizophrenia according to the Diagnostic and Statistical Manual of Mental Disorder IV (DSM-IV) criteria for schizophrenia by at least two experienced senior psychiatrists; (2) age from 18 to 55 years; (3) underwent a standardized battery of examinations, including family history, extensive drug and alcohol assessment, physical and neurological examination, and laboratory tests to exclude substance-induced psychotic disorders or psychosis caused by general medical condition. 1035 healthy controls were randomly selected from the Health Examination Center of the Affiliated Hospital of Guangdong Medical University. Based on unstructured interviews and physical examination reports, healthy controls with a personal or family history of psychiatric disorders, substance abuse and serious somatic illnesses were excluded. All subjects were Han Chinese origin.

### Symptom and Neurocognitive Function Assessment

The psychotic symptoms of patients with schizophrenia were evaluated with the Positive and Negative Symptom Scale (PANSS). Although the items of the PANSS are divided into three subscales (the positive, negative and general psychopathology scale), several factor analyses have shown that five-factor models better characterize PANSS data (Van et al., 2006). Thus, our results for the PANSS were presented in a five-factor model encompassing the following factors: positive (total score of P1, P5, P6, G9), negative (total score of N1, N2, N3, N4, N6, G16), excitement (total score of P4, P7, G4, G14), depression/anxiety (total score of G1, G2, G3, G6, G15), and cognitive (total score of P2, N5, G5, G10, G11) (Lindenmayer et al., 1994). Additionally, the neurocognitive function of the patients was assessed using the Brief Assessment of Cognition in Schizophrenia (BACS), as described previously (Ma et al., 2014).

### DNA Extraction and Genotyping

Genomic DNA from EDTA-treated peripheral blood was extracted using the TIANamp Blood DNA Kit (Tiangen Biotech, Beijing, People’s Republic of China). The improved multiplex ligation detection reaction (iMLDR) method (Genesky Biotechnologies Inc., Shanghai, China) was used to genotype candidate SNPs, as described previously (Xu et al., 2018). The primer information for the multiplex polymerase chain reaction (PCR) is described in Supplementary Table S1.

### Statistical Analysis

The statistical analysis was performed using SPSS 22.0 software. The descriptive variables are presented as the mean ± standard deviation (SD). *P* < 0.05 was considered significant for all statistical tests. Hardy–Weinberg equilibrium was tested using Pearson’s chi-square (χ^2^) test. The allelic and genotypic frequencies were compared between patients and controls using χ^2^ tests. Generalized odds ratios (ORs) with 95% confidence intervals (CIs) of the alleles were calculated. Subjects’ basic demographic data, such as age, gender, and family history, were measured using χ^2^ tests. To test the effect of genotype on phenotypes, analysis of variance (ANOVA) was conducted with the genotype as the fixed factor, and the five factors (positive, negative, excitement, depression/anxiety, and cognitive) from the PANSS, age, age at onset, duration and cognitive scores (the BACS total and 5 index scores) were the dependent factors. Multiple test corrections were conducted by Bonferroni’s test. Power calculations were performed using QUANTO 1.2 software. The LD status was determined using the Haploview 4.2 program. Only those haplotypes with frequencies greater than 3% were further analyzed.

## Results

### Demographic Characteristics

The patients and controls had comparable age and gender distributions in the TCF4 polymorphisms (all *P* > 0.05), as described in Supplementary Table S2. In addition, for the analysis of TCF4 SNPs effect in clinical characteristics, we found no difference in duration, family history and age at onset between the genotypes of selected SNPs (shown in Table 2).

### Association Study of SNPs (rs9960767, rs2958182, rs4309482, rs12966547) and Schizophrenia

A total of 1137 patients and 1035 controls were genotyped for rs4309482, rs12966547 and rs9960767. 1916 samples (1021 patients and 895 controls) were genotyped for rs2958182. In results, only AA and CA were present in rs9960767, and there were 6 CA genotypes in schizophrenia patients and 2 in controls; thus, rs9960767 could not be included in the statistics. The distributions of the TCF4 rs2958182, rs4309482, and rs12966547 polymorphisms in our cohort are shown in Table 1. The frequency distribution of each tag SNP (rs2958182, rs4309482, rs12966547) in the case group and the controls was in Hardy–Weinberg equilibrium (all *P* > 0.05). No significant differences were found in the frequencies of genotypes (χ^2^ = 2.15, *P* = 0.34 for rs2958182; χ^2^ = 2.30, *P* = 0.32 for rs4309482; χ^2^ = 2.46, *P* = 0.29 for rs12966547) or alleles (*P* = 0.50, OR = 0.94, 95% CI: 0.79–1.12 for rs2958182; *P* = 0.21, OR = 1.08, 95% CI: 0.96–1.08 for rs4309482; *P* = 0.20, OR = 0.92, 95% CI: 0.82–1.04 for rs12966547) between the patients with schizophrenia and the controls. Additionally, in the gender-stratified analysis, there were no significant differences in either genotype or allele distributions between schizophrenic patients and controls (Supplementary Table S3).

**Table 1:** Genotype and allele frequencies of TCF4 gene rs2958182, rs4309482, and rs12966547 polymorphisms in schizophrenic patients and controls.

	N	Genotype N (%)			χ^2^	*P*	Allele N (%)		χ^2^	*P*	OR	95%CI	HWE χ^2^	*P*
**rs2958182**		**AA**	**AT**	**TT**			**A**	**T**						
Patients	1021	33 (3.23%)	254 (24.88%)	734 (71.89%)	2.15	0.34	320 (15.67%)	1722 (84.33%)	0.46	0.50	0.94	0.79–1.12	0.01	0.94
Controls	895	24 (2.68%)	247 (27.60%)	624 (69.72%)			295 (16.48%)	1495 (83.52%)						
**rs4309482**		**GG**	**GA**	**AA**			**G**	**A**						
Patients	1137	450 (39.58%)	534 (46.97%)	153 (13.46%)	2.30	0.32	1434 (63.06%)	840 (36.94%)	1.58	0.21	1.08	0.96–1.08	1.98	0.16
Controls	1035	377 (36.43%)	513 (49.57%)	145 (14.01%)			1267 (61.21%)	803 (38.79%)						
**rs12966547**		**GG**	**GA**	**AA**			**G**	**A**						
Patients	1137	154 (13.54%)	534 (46.97%)	449 (39.49%)	2.46	0.29	842 (37.03%)	1432 (62.97%)	1.68	0.20	0.92	0.82**–**1.04	2.04	0.15
Controls	1035	146 (14.11%)	514 (49.66%)	375 (36.23%)			806 (38.94%)	1264 (61.06%)						


*OR: odds ratio; 95%CI: 95% confidence interval; HWE: Hardy–Weinberg equilibrium.*

### TCF4 SNPs and Clinical Characteristics

The genotype distributions of the three SNPs were in Hardy–Weinberg equilibrium (all *P* > 0.05, data not shown). The difference was significant among genotypes of rs4309482 in the positive scores (*F* = 3.34, *P* = 0.04) and excitement scores (*F* = 3.27, *P* = 0.04) and genotypes of rs12966547 in positive scores (*F* = 3.57, *P* = 0.03), However, three positive results did not pass the Bonferroni corrections (*P* = 0.12, *P* = 0.12, *P* = 0.09, respectively). There was no significant difference in other items in rs4309482 and rs12966547. Furthermore, there was no significant difference in age at onset, family history, duration, and total or five-factor scores for the PANSS and BACS scores when comparing the different rs2958182 genotypes (Table 2 and Supplementary Table S4).

**Table 2:** Genotypes of TCF4 gene polymorphisms and clinical characteristics of schizophrenic patients.

Variables	N	Genotype			Statistic tests	*P*	*P^c^*
rs2958182		AA	AT	TT			
Age (Mean ± SD, year)	30/241/698	36.17 ± 13.77	34.05 ± 13.34	35.04 ± 13.81	*F* = 0.61	0.54	
Family history: n (%)							
+	146	3 (2)	32 (21.9)	111 (76)	χ^2^ = 1.73	0.42	
–	875	30 (3.4)	222 (25.3)	623 (71.2)			
Duration (Mean ± SD, Month)	30/240/696	179.4 ± 200.6	174.1 ± 196.8	167.9 ± 199.0	*F* = 0.12	0.88	
Age at onset (Mean ± SD, year)	30/241/696	27.43 ± 11.01	24.86 ± 9.97	25.32 ± 10.16	*F* = 0.89	0.41	
**PANSS**							
Positive	30/241/698	11.93 ± 4.57	13.37 ± 5.35	13.73 ± 4.82	*F* = 2.21	0.11	
Negative	30/241/698	14.53 ± 5.59	16.39 ± 8.78	16.72 ± 8.2	*F* = 1.09	0.34	
Excitement	30/241/698	10.47 ± 3.80	9.88 ± 4.45	9.99 ± 4.53	*F* = 0.23	0.79	
Depression/Anxiety	30/241/698	8.13 ± 3.31	7.91 ± 3.58	8.07 ± 3.49	*F* = 0.19	0.82	
Cognitive	30/241/698	9.77 ± 3.25	9.99 ± 4.04	10.31 ± 4.01	*F* = 0.75	0.47	
							
**rs4309482**		**AA**	**GA**	**GG**			
Age (Mean ± SD, year)	145/514/425	33.97 ± 13.43	35.13 ± 13.91	34.11 ± 13.20	*F* = 0.83	0.44	
Family history: n (%)							
+	153	20 (13)	72 (47)	61 (39.8)	χ^2^ = 0.02	0.99	
–	984	133 (13.5)	462 (46.9)	389 (39.5)			
Duration (Mean ± SD, Month)	144/512/424	141.8 ± 206.7	163.2 ± 202.8	144.8 ± 177.4	*F* = 1.33	0.27	
Age at onset (Mean ± SD, year)	145/513/423	25.21 ± 9.15	25.48 ± 10.25	24.81 ± 9.82	*F* = 0.53	0.59	
PANSS							
Positive	145/514/425	14.44 ± 5.24	13.26 ± 4.88	13.6 ± 4.82	*F* = 3.34	0.04^∗^	0.12
Negative	145/514/425	17.84 ± 8.55	16.13 ± 8.15	16.91 ± 8.12	*F* = 2.77	0.06	
Excitement	145/514/425	10.1 ± 4.38	10.12 ± 4.55	9.42 ± 4.13	*F* = 3.27	0.04^∗^	0.12
Depression/Anxiety	145/514/425	8.86 ± 4.11	8.14 ± 3.53	8.32 ± 3.65	*F* = 2.16	0.12	
Cognitive	145/514/425	11.24 ± 4.65	10.33 ± 4.20	10.4 ± 3.78	*F* = 2.92	0.05	
							
**rs12966547**		**GG**	**GA**	**AA**			
Age (Mean ± SD, year)	146/514/423	33.9 ± 13.40	35.12 ± 13.92	34.18 ± 13.19	*F* = 0.78	0.46	
Family history: *n* (%)							
+	153	20 (13)	72 (47)	61 (39.8)	χ^2^ = 0.04	0.98	
–	984	134 (13.6)	462 (46.9)	388 (39.4)			
Duration (Mean ± SD, Month)	145/512/422	140.9 ± 206.3	163.1 ± 202.8	145.4 ± 177.6	*F* = 1.31	0.27	
Age at onset (Mean ± SD, year)	146/513/421	25.21 ± 9.12	25.48 ± 10.25	24.84 ± 9.83	*F* = 0.47	0.62	
**PANSS**							
Positive	146/514/423	14.45 ± 5.22	13.23 ± 4.89	13.61 ± 4.79	*F* = 3.57	0.03^∗^	0.09
Negative	146/514/423	17.76 ± 8.58	16.2 ± 8.20	16.84 ± 8.06	*F* = 2.23	0.11	
Excitement	146/514/423	10.06 ± 4.39	10.12 ± 4.55	9.45 ± 4.12	*F* = 2.95	0.05	
Depression/Anxiety	146/514/423	8.83 ± 4.11	8.15 ± 3.53	8.31 ± 3.66	*F* = 1.98	0.14	
Cognitive	146/514/423	11.2 ± 4.67	10.36 ± 4.20	10.38 ± 3.77	*F* = 2.6	0.08	


*PANSS: positive and negative symptom scale, ^∗^P < 0.05, P^c^: the P value corrected by Bonferroni correction.*

### LD Analysis

We performed an LD analysis of 4 loci. Strong LD was observed between rs4309482 and rs12966547 (*D’* = 1.0, *r*^2^= 0.997). rs2958182 had LD with rs9960767 (*D’* = 1.0, *r*^2^= 0.0); however, neither is strongly in LD with rs4309482 (*D’* = 0.04, *r*^2^= 0.0) or rs12966547 (*D’* = 0.04, *r*^2^ = 0.001). In addition, rs9960767 also showed LD with rs4309482 (*D’* = 0.51, *r*^2^= 0.001) and rs12966547 (*D’* = 0.51, *r*^2^= 0.001) (Supplementary Table S5).

## Discussion

The TCF4 gene was highly associated with schizophrenia in a recent GWAS analysis (Schizophrenia Psychiatric Genome-Wide Association Study [GWAS] Consortium, 2011). As a member of the bHLH group of proteins, TCF4 controls critical steps of various developmental and possibly plasticity-related transcriptional programs in the central nervous system (Quednow et al., 2014). Increasing compelling evidence supports the crucial role of TCF4 during neurodevelopment and raises the possibility that TCF4 genetic perturbations may increase the risk for schizophrenia. In the present case-control study, we evaluated the potential association of the four SNPs (rs9960767 rs2958182, rs4309482, rs12966547) of the TCF4 gene with schizophrenia in a sample of 1137 unrelated patients with schizophrenia and 1035 unrelated healthy people. We further aimed to test the effects of a genetic risk variant on phenotype and to identify the genetic sources of phenotypic heterogeneity.

**FIGURE 1:**
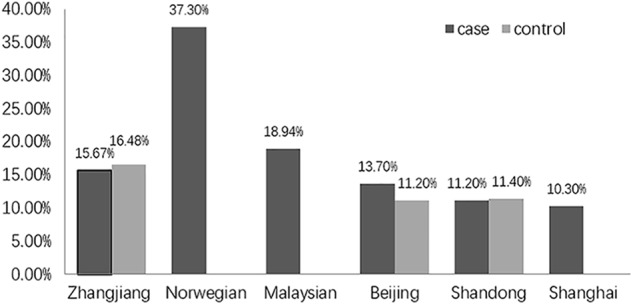
The frequency of the rs2958182 A-allele in different studies.

Unexpectedly, we found no association of the genotype or allele distribution of rs2958182 with schizophrenia in our sample from Zhanjiang. This result is unexpected because the susceptibility of this SNP to schizophrenia has been repeatedly found in previous studies. The inconsistent results might be due to ethnic and geographic heterogeneity. In the present study, the frequency of the rs2958182 A allele detected in our cohort from China (15.67% in the cases and 16.48% in the controls) was lower than that previously observed in other ethnicities, including Norwegians (37.3% in the cases) (Wirgenes et al., 2012) and Malaysians (18.94% in the cases) (Chow et al., 2016). In the same ethnicity, genetic variation in TCF4 also exists among geographic groups within the Chinese Han population. The A allele frequency of rs2958182 in our cohort from Zhanjiang, in Southern China, was higher than that in the Chinese cohort of Beijing (13.7% in the cases and 11.2% in the controls) (Hui et al., 2015), Shandong (11.2% in the cases and 11.4% in the controls) (Zhu et al., 2013) and Shanghai (10.3% in the cases) (Li et al., 2010) (as shown in Figure 1). Furthermore, we failed to find any association of rs2958182 with clinical neurocognitive characteristics of schizophrenia, which also might be due to the bias in genotype frequencies. In addition to geographical and ethnic factors, the case collection process, diagnostic criteria and environmental factors may also explain these differences. Therefore, rs2958182 susceptibility in schizophrenia should be further verified in different populations. In addition, with our study sample and assuming a risk allele frequency of 15.67%, we had 86.7% power to detect a genotype relative risk with an odds ratio of 1.3 at the 0.05 level. Therefore, there is still a 13.3% chance of a type II error. A false negative result cannot be excluded.

The variant rs9960767 was a common polymorphic variant and was proved to be a risk allele for schizophrenia in populations of European origin (Quednow et al., 2011; Steinberg et al., 2011) and in the Canadian population (Albanna et al., 2014). However, in China, no polymorphic variation in rs9960767 was reported in a large sample (2496 schizophrenia cases and 5184 control subjects) from East China in a previous study (Li et al., 2010). In our Southern Chinese sample, we obtained rare frequency mutations in rs9960767, with only 6 CA genotypes in patients with schizophrenia and 2 CA genotypes in controls. The frequency of the CA genotype was 0.53% in patients and 0.19% in controls. Both of these frequencies are significantly lower than those in the HapMap HCB data (CA = 2.33%)^1^. These results suggested that ethnic heterogeneity also exists in rs9960767. Furthermore, the CC haplotype absent in rs9960767 and different allele frequencies lead to the heterogeneity in LD to rs2958182 and rs9960767. Although rs2958182 is a marker near (∼6 kb) and in complete LD with rs9960767 in people of European origin, in our sample, the LD analysis showed **D’** = 1, **r**^2^ = 0. The dramatic results in our study highlight the inherent problems of LD index **D’** and **r**^2^. **D’** is not a sensitive measure of LD for rare mutations such as rs9960767. However, **r**^2^, which summarizes both recombination and mutational history values, is better able to assess LD. This **r**^2^ value indicated the lack of LD observed for rs2958182 and rs9960767 in our Chinese sample. Therefore, it is of particular practical importance to take great care in case-control studies that evaluate the risk of two associations for schizophrenia in a Chinese sample.

Our results indicated that rs4309482 and rs12966547 are in strong LD in our sample of Southern Chinese Han individuals. The rs4309482 and rs12966547 genotypes were associated with positive symptoms and excitement symptoms using a five-factor model of the PANSS, but not survived in the multiple test correction. In previous studies, these two variants have been linked to several psychosis phenotypes. In a larger Norwegian study sample, these two risk alleles were confirmed to be related to poorer verbal fluency (Wirgenes et al., 2012). Furthermore, rs12966547 of TCF4 was significantly associated with earlier age at onset in a Malaysian population (Chow et al., 2016). However, in a German study, rs4309482 was not associated with schizophrenia, including genotypes, alleles, clinical symptoms and cognitive function (Papiol et al., 2011). Although rs4309482 and rs12966547 of TCF4 appear to be risk factors influencing the phenotypes in schizophrenia, it seems unlikely that a SNP could account for a disorder as complex as schizophrenia. The mechanisms underlying the effect of SNPs of TCF4 in schizophrenia deserve further exploration.

In previous studies, TCF4 has been linked to cognitive functions (Wirgenes et al., 2012; Zhu et al., 2013; Albanna et al., 2014; Hui et al., 2015). Wirgenes et al. (2012) reported that risk alleles of rs4309482 are associated with poorer executive function in the form of verbal fluency in Norwegian population. Our data showed marginally significant differences in cognitive domain of Reasoning and Problem Solving between the genotypes of rs4309482, but not survived in the multiple test correction. The similar results were found in the cognitive subscale of PANSS (shown in Supplementary Table S3). It is the first time to analyze the effect of rs4309482 in cognitive impairment of schizophrenia in Chinese population, which needs to be further confirmed.

In conclusion, in Southern Chinese cohort, we failed to find the associations of SNPs (rs4309482 and rs12966547) in TCF4 to schizophrenia and failed to replicate the association of rs2958182 to schizophrenia found in east Chinese cohort. These findings argue against the rs2958182 polymorphism being a risk factor for schizophrenia in the Chinese population. More SNPs, which suggested by GWAS in TCF4 should be included in the further research for insights to the risk of TCF4 in schizophrenia.

## Ethics Statement

This study was carried out in accordance with the recommendations of “the Affiliated Hospital of Guangdong Medical University, the Ethical Committee of the Affiliated Hospital of Guangdong Medical University” with written informed consent from all subjects. All subjects gave written informed consent in accordance with the Declaration of Helsinki. The protocol was approved by the “Ethical Committee of the Affiliated Hospital of Guangdong Medical University.”

## Author Contributions

KL, GM, BZ, JL, and YW supervised the entire project and gave critical comments on the manuscript. DL, XL, JF, HaY, and ZL contributed to the data collection. HuY, CL, SX, XZ, and XW participated in genetic analyses. ZD and XS administered the neuropsychological tests. JY, DZ, and YL managed the literature searches, collected the data, undertook the statistical analyses, and wrote the draft of the manuscript. All authors approved the final manuscript.

## Conflict of Interest Statement

The authors declare that the research was conducted in the absence of any commercial or financial relationships that could be construed as a potential conflict of interest.

## Footnotes

1 https://www.ncbi.nlm.nih.gov/projects/SNP/snp_ref.cgi?rs=9960767
