# Supplementary material for: Genetic Variability of TCF4 in Schizophrenia of Southern Chinese Han Population: A Case-Control Study
Source: Front Genet. 2019 May 28;10:513. doi: 10.3389/fgene.2019.00513 (PMC6546831; doi:10.3389/fgene.2019.00513)
Supplement: Supplementary file 1 [file Table_1.DOCX]

Supplementary Material

**Genetic Variability of TCF4 in Schizophrenia of Southern Chinese Han Population: a Case-control Study**

**Jingwen Yin^1#^, Dongjian Zhu^1#^, You Li^2, 3#^，Dong Lv^1^，Huajun Yu^4^，Chunmei Liang^2, 3^, Xudong Luo^1^, Xusan Xu^3^, Jiawu Fu^2^, Haifeng Yan^1^, Zhun Dai^1^, Xia Zhou^3^, Xia Wen^3^, Susu Xiong^1^, Zhixiong Lin^1^, Juda Lin^1^, Bin Zhao^3^, Yajun Wang^5*^, Keshen Li ^3, 6, 7*^, Guoda Ma^2, 3*^**

*** Correspondence:**Yajun Wang: wangyajuny1977@aliyun.com

Keshen Li: keshenli1971@163.com

Guoda Ma: [sihan1107@126.com](mailto:sihan1107@126.com)

# Table S1 The primers of four SNPs for the multiplex PCR reaction

| SNP | Primer |
| --- | --- |
| rs9960767 Forward primer | CCCAGCAAGAGATTCCATTGTATGCT |
| rs9960767 Reverse primer | TGTGGCTTTAACAGGGGCTTTTGA |
| rs9960767FA | TGTTCGTGGGCCGGATTAGTTTGATTTGAAGCATAAAAATTTCTATGAGATGCAA |
| rs9960767FC | TCTCTCGGGTCAATTCGTCCTTTTGATTTGAAGCATAAAAATTTCTATGAGATGTAC |
| rs9960767FP | TTCACAAATTATTACCCCTTTAAAATGTAAAACTTTTTTTTTTTTTTTTTT |
| rs2958182 Forward primer | CCCTGACTCCTAGACGTCCAATTC |
| rs2958182 Reverse primer | CCTTGGTGAGGACAGGTGTATGAA |
| rs2958182FA | TCTCTCGGGTCAATTCGTCCTTACATTTTGGACCTATGTCCTTCCAAGACTA |
| rs2958182FP | ATCCCTTACAATGTCTCAAGAAATAGACTTATGTTTTTTTTTTT |
| rs2958182FT | TGTTCGTGGGCCGGATTAGTACATTTTGGACCTATGTCCTTCCAAGACTT |
| rs4309482 Forward primer | ACGATGTGTATAAATCTCCAAAGGCAT |
| rs4309482 Reverse primer | GGACTCTGAGCAGTTCCATCGCT |
| rs4309482FA | TACGGTTATTCGGGCTCCTGTGGCATCATGCTAAGTGACAGGAGACA |
| rs4309482FG | TTCCGCGTTCGGACTGATATGGCATCATGCTAAGTGACAGGAGACG |
| rs4309482FP | GTCTCAAAAAGTTACATACCGTGTGATTCTTTTTTTTTTTTT |
| rs12966547 Forward primer | GGTCTTGGAAGGAATAGACACAAGCT |
| rs12966547 Reverse primer | TGGTGGAGTTGAAACAGAGACCTC |
| rs12966547FA | TACGGTTATTCGGGCTCCTGTAAAAAATAAAAGTAAATAACATTTAATAGGACACGA |
| rs12966547FG | TTCCGCGTTCGGACTGATATAAAAAATAAAAGTAAATAACATTTAATAGGACATGG |
| rs12966547FP | ATAGTAAGTCAGAGTGGACTTTTCATTRACTCTTTTTTTTTTTTTTTTTT |

# Table S2 The demographic characteristics of SNPs in TCF4 of schizophrenic patients and controls

| Variables |  | Schizophrenic patients | Controls | Statistical tests |
| --- | --- | --- | --- | --- |
| rs2958182 |  | N = 1021 | N = 895 |  |
| Mean Age ± SD (year) |  | 34.89 ± 13.79 | 34.93 ± 9.84 | t = 0.09, *P* = 0.93 |
| Gender n (%) | Male | 645(0.63) | 533(0.60) | χ2= 2.64,  *P* = 0.10 |
|  | Female | 376(0.37) | 362(0.40) |  |
|  | | | | |
| Rs9960767, rs4309482,  rs12966547 |  | N = 1137 | N = 1035 |  |
| Mean Age ± SD (year) |  | 34.65 ± 13.66 | 34.31 ± 9.44 | t = 0.68, *P* = 0.50 |
| Gender n (%) | Male | 725(0.64) | 618(0.60) | χ2 = 3.77, *P* = 0.06 |
|  | Female | 412(0.36) | 417(0.40) |  |

# Table S3 Gender-stratified Genotype and allele frequencies of TCF4 gene rs2958182,rs4309482

# and rs12966547 polymorphisms in schizophrenic patients and controls

|  | N | Genotype n (%) | | | χ2 | *P* | Allele n (%) | | χ2 | *P* | OR | 95%CI | χ2^HWE^ | *P^HWE^* |
| --- | --- | --- | --- | --- | --- | --- | --- | --- | --- | --- | --- | --- | --- | --- |
| rs2958182 | | AA | AT | TT |  |  | A | T |  |  |  |  |  |  |
| **Male** |  |  |  |  |  |  |  |  |  |  |  |  |  |  |
| Patients | 645 | 23(3.57%) | 173(26.82%) | 449(69.61%) | 0.14 | 0.93 | 219(16.98%) | 1071(83.02%) | 0.01 | 0.91 | 1.01 | 0.82-1.26 | 0.37 | 0.54 |
| Controls | 533 | 17(3.19%) | 145(27.20%) | 371(69.61%) |  |  | 179(16.79%) | 887(83.21%) |  |  |  |  |  |  |
| **Female** |  |  |  |  |  |  |  |  |  |  |  |  |  |  |
| Patients | 376 | 10(2.66%) | 81(21.54%) | 285(75.80%) | 4.58 | 0.10 | 101(13.43%) | 651(86.57%) | 1.98 | 0.16 | 0.81 | 0.61-1.09 | 0.80 | 0.37 |
| Controls | 362 | 7(1.93%) | 102(28.18%) | 253(69.89%) |  |  | 116(16.02%) | 608(83.98%) |  |  |  |  |  |  |
|  |  |  |  |  |  |  |  |  |  |  |  |  |  |  |
| rs4309482 | | GG | GA | AA |  |  | G | A |  |  |  |  |  |  |
| **Male** |  |  |  |  |  |  |  |  |  |  |  |  |  |  |
| Patients | 725 | 293(40.42%) | 336(46.34%) | 96(13.24%) | 2.68 | 0.26 | 922(63.59%) | 528(36.41%) | 2.27 | 0.13 | 1.13 | 0.96-1.32 | 0.76 | 0.38 |
| Controls | 618 | 223(36.08%) | 305(49.35%) | 90(14.56%) |  |  | 751(60.76%) | 485(39.24%) |  |  |  |  |  |  |
| **Female** |  |  |  |  |  |  |  |  |  |  |  |  |  |  |
| Patients | 412 | 157(38.11%) | 198(48.06%) | 57(13.83%) | 0.28 | 0.87 | 512(62.14%) | 312(37.86%) | 0.01 | 0.91 | 1.01 | 0.83-1.23 | 1.36 | 0.24 |
| Controls | 417 | 154(36.93%) | 208(49.88%) | 55(13.19%) |  |  | 516(61.87%) | 318(38.13%) |  |  |  |  |  |  |
|  |  |  |  |  |  |  |  |  |  |  |  |  |  |  |
| rs12966547 | | GG | GA | AA |  |  | G | A |  |  |  |  |  |  |
| **Male** |  |  |  |  |  |  |  |  |  |  |  |  |  |  |
| Patients | 725 | 97(13.38%) | 336(46.34%) | 292(40.28%) | 2.69 | 0.26 | 530(36.55%) | 920(63.45%) | 2.18 | 0.14 | 0.89 | 0.76-1.04 | 0.88 | 0.35 |
| Control | 618 | 90(14.56%) | 306(49.51%) | 222(35.92%) |  |  | 486(39.32%) | 750(60.68%) |  |  |  |  |  |  |
| **Female** |  |  |  |  |  |  |  |  |  |  |  |  |  |  |
| Patients | 412 | 57(13.83%) | 198(48.06%) | 157(38.11%) | 0.28 | 0.87 | 312(37.86%) | 512(62.14%) | 0.05 | 0.83 | 0.98 | 0.18-1.19 | 1.25 | 0.26 |
| Controls | 417 | 56(13.43%) | 208(49.88%) | 153(36.69%) |  |  | 320(38.37%) | 514(61.63%) |  |  |  |  |  |  |

OR: odds ratio; 95%CI: 95% confidence interval; HWE: Hardy-Weinberg equilibrium.

# Table S4 Analysis of neurocognitive function scores of three SNPs in TCF4

| Variables | N | Genotype | | | F | *P* |
| --- | --- | --- | --- | --- | --- | --- |
| rs2958182 |  | AA | AT | TT |  |  |
| Working memory | 15/73/246 | 13.8 ± 8.50 | 16.58 ± 9.07 | 14.76 ± 9.61 | 1.19 | 0.31 |
| Semantic fluency | 14/78/251 | 34.21 ± 13.55 | 30.46 ± 12.98 | 28.98 ± 12.56 | 1.40 | 0.25 |
| Letter fluency | 14/75/241 | 8.86 ± 4.07 | 10.01 ± 6.10 | 9.6 ± 6.07 | 0.27 | 0.77 |
| Verbal memory | 13/70/236 | 27.62 ± 10.77 | 24.01 ± 16.54 | 21.27 ± 12.85 | 2.18 | 0.12 |
| Motor speed | 15/77/248 | 44.27 ± 7.91 | 45.43 ± 13.74 | 43.4 ± 11.73 | 0.84 | 0.43 |
| Reasoning and problem solving | 13/66/223 | 10.08 ± 6.26 | 8.82 ± 7.79 | 7.41 ± 6.16 | 1.97 | 0.14 |
| Attention and processing speed | 12/68/222 | 21.42 ± 15.64 | 23.18 ± 14.27 | 21.41 ± 12.77 | 0.47 | 0.63 |
|  |  | | | | | |
| rs4309482 |  | AA | GA | GG |  |  |
| Working memory | 55/192/177 | 16.62 ± 9.21 | 15.1 ± 9.37 | 14.69 ± 9.13 | 0.91 | 0.40 |
| Semantic fluency | 56/195/182 | 30.02 ± 12.56 | 28.29 ± 12.73 | 27.67 ± 12.52 | 0.74 | 0.48 |
| Letter fluency | 54/186/179 | 9.09 ± 5.75 | 9.37 ± 5.96 | 9.23 ± 5.75 | 0.06 | 0.95 |
| Verbal memory | 53/181/174 | 23.32 ± 12.38 | 23.2 ± 15.29 | 21.06 ± 13.33 | 1.17 | 0.31 |
| Motor speed | 56/196/177 | 50.45 ± 15.19 | 47.29 ± 17.14 | 48.81 ± 17.86 | 0.85 | 0.43 |
| Reasoning and problem solving | 53/177/161 | 5.7 ± 5.28 | 8.12 ± 6.92 | 7.5 ± 5.87 | 3.02 | 0.05 |
| Attention and processing speed | 51/179/160 | 20.59 ± 12.07 | 20.41 ± 13.82 | 22.44 ± 13.31 | 1.04 | 0.36 |
|  |  | | | | | |
| rs12966547 |  | GG | GA | AA |  |  |
| Working memory | 56/192/176 | 16.68 ± 9.14 | 15.07 ± 9.36 | 14.7 ± 9.15 | 0.98 | 0.38 |
| Semantic fluency | 57/195/181 | 30.11 ± 12.46 | 28.27 ± 12.72 | 27.66 ± 12.55 | 0.82 | 0.44 |
| Letter fluency | 55/186/178 | 9.18 ± 5.74 | 9.35 ± 5.95 | 9.23 ± 5.76 | 0.03 | 0.97 |
| Verbal memory | 54/181/173 | 23.8 ± 12.75 | 23.1 ± 15.18 | 21.01 ± 13.34 | 1.32 | 0.27 |
| Motor speed | 57/196/176 | 50.51 ± 15.06 | 47.25 ± 17.14 | 48.83 ± 17.91 | 0.92 | 0.40 |
| Reasoning and problem solving | 54/177/160 | 5.89 ± 5.42 | 8.06 ± 6.89 | 7.51 ± 5.89 | 2.46 | 0.09 |
| Attention and processing speed | 52/179/159 | 21.06 ± 12.42 | 20.36 ± 13.74 | 22.35 ± 13.31 | 0.94 | 0.39 |

# Table S5 Estimation of LD between each pair of loci in TCF4

| SNP D'(r2) | rs2958182 | rs9960797 | rs12966547 | rs4309482 |
| --- | --- | --- | --- | --- |
| rs2958182 | -- | -- | -- | -- |
| rs9960767 | 1.0(0.00) | -- | -- | -- |
| rs12966547 | 0.04(0.001) | 0.51(0.001) | -- | -- |
| rs4309482 | 0.04(0.00) | 0.51(0.001) | 1.0(0.997) | -- |
|  | | | | |
